# Temporal trends and disparities in sudden cardiac death among colorectal cancer patients: A nationwide study

**DOI:** 10.18632/oncoscience.635

**Published:** 2025-11-07

**Authors:** Eric Sanji, Juste Niba, Terence Longla, Lathifa Nnap, Charmain Mbaki, Bonaventure Dzekem

**Affiliations:** ^1^Magnolia Regional Health Center, Corinth, MS 38834, USA; ^2^Swiss Care Hospital, Limbe, Cameroon; ^3^Woodhull Medical Center, Brooklyn, NY 11206, USA; ^4^Faculty of Health Sciences, University of Buea, Southwest Region, Cameroon; ^5^Cygnet Wast Hills Hospital, Birmingham, United Kingdom; ^6^Department of Medicine, University of Chicago, Chicago, IL 60637, USA

**Keywords:** racial disparities, sudden cardiac death, colorectal cancer, cancer mortality, cardio-oncology

## Abstract

Background: Colorectal cancer (CRC) patients are at risk of cardiovascular problems, especially sudden cardiac death, due to aging, pre-existing comorbidities, and cardiotoxic medicines. Few large-scale epidemiologic studies on SCD trends and disparities in CRC patients exist. The goal is to examine US CRC decedent SCD trends and sociodemographic variations from 1999 to 2020.

Methods: A retrospective population-based analysis was conducted using the CDC WONDER Multiple Cause of Death database (1999–2020). Colorectal cancer (CRC) fatalities were identified using ICD-10 codes C18–C21, and sudden cardiac death (SCD) was defined using ICD-10 codes I46.1, I46.9, R96.0, I49.0, and I21–I24. Age-adjusted and crude death rates were estimated by sex, race/ethnicity, age group, and U.S. state. Temporal trends were assessed using linear regression. Subgroup analyses were also performed by age, sex, and geographic region.

Results: The age-adjusted mortality rate of SCD among CRC decedents reduced from 1.2 to 0.5 per 100,000 population between 1999 and 2020, demonstrating a steady trend. Males had greater SCD rates than females for two decades. Age-stratified analysis showed that CRC patients aged 65–84 carried the most SCD burden. Race and ethnicity affected SCD mortality, with Black and Asian/Pacific Islanders dying more than Whites. Geographic study found high SCD rates in Nebraska and Vermont and low rates in California and Texas.

Conclusions: Despite age-adjusted rate decreases over two decades, SCD remains a significant contributor to death in CRC patients. Persistent discrepancies by gender, race, and geography underline the importance of individualized cardio-oncology surveillance, equitable preventative initiatives, and focused public health interventions.

## INTRODUCTION

CRC is one of the most common malignancies and a primary cause of cancer-related deaths globally, with millions of new cases and deaths annually [1, 2]. Over the past few decades, screening methods (e.g., colonoscopy, stool-based tests), early detection methods, and multimodal therapeutic strategies (including surgery, chemotherapy, radiation, and targeted molecular therapies) have greatly improved CRC patient survival rates, but a growing body of evidence highlights the persistent and evolving burden of non-cancer-related mortality in this population [3, 4]. In long-term cancer patients, cardiovascular problems are a leading cause of death, emphasizing the need to understand cardiotoxicity and cardiovascular risk in this vulnerable group [5, 6]. Sudden cardiac death (SCD) is a serious, unexpected, and severe cardiovascular condition that requires epidemiological study.

SCD in cancer patients is a complex phenomenon impacted by a variety of factors specific to both the underlying disease and its therapy. Pre-existing cardiovascular comorbidities (such as hypertension, diabetes, and dyslipidemia), direct cardiotoxic effects of various cancer therapies (e.g., anthracyclines, HER2-targeted agents, fluoropyrimidines, and chest radiation), indirect cardiotoxicity through electrolyte imbalances or autonomic dysfunction, cancer-related inflammation, and a prothrombotic state often induced by the malignancy itself [7–9] are among these factors. Targeted therapies like epidermal growth factor receptor (EGFR) inhibitors, used in metastatic CRC, can cause electrolyte disturbances, particularly hypomagnesemia, which can prolong QT interval and increase the risk of fatal arrhythmias and sudden cardiac death [10]. For CRC patients, in particular, the intersection of an increasingly aging patient demographic (who inherently carry a higher burden of cardiovascular risk factors), the physiological stress of the disease, and the potential cardiotoxicity of various treatments commonly used in CRC management (e.g., 5-fluorouracil, oxaliplatin, bevacizumab) poses a significant risk for adverse cardiac events, including fatal arrhythmias culminating in SCD [11, 12].

Despite the well-known and growing field of cardio-oncology, which seeks to address cancer patients’ cardiovascular health, there are fewer comprehensive, large-scale, long-term epidemiological analyses focusing on the incidence and demographic trends of SCD among CRC patients in the United States than studies on other cancer types or general cardiovascular populations. Understanding the changing trends, persistent demographic disparities, and potential risk factors associated with SCD in this vulnerable population is critical for informing clinical practice, developing targeted preventive strategies, optimizing cardiac surveillance protocols during and after cancer treatment, and, ultimately, improving overall survival and long-term quality of life for the growing population of CRC survivors [13, 14].

This study seeks to provide a full and current epidemiological investigation of sudden cardiac death rates among colorectal cancer patients in the United States over a 21-year period (1999–2020). We hope to assess changes in SCD mortality across different age groups, sexes, and racial/ethnic categories by utilizing robust national mortality data. Through this investigation, we hope to elucidate the burden of SCD in this critical cohort, identify persistent disparities that may indicate inequities in care or underlying biological predispositions, and contribute to a more comprehensive understanding of cardiovascular outcomes in CRC survivors. Our findings will be meticulously compared to current U.S. and global literature to contextualize observed patterns and disparities, giving useful insights for both clinical practice and public health initiatives aimed at improving the long-term health of colon cancer patients.

## RESULTS

### Sociodemographic data

**Figure 1:**
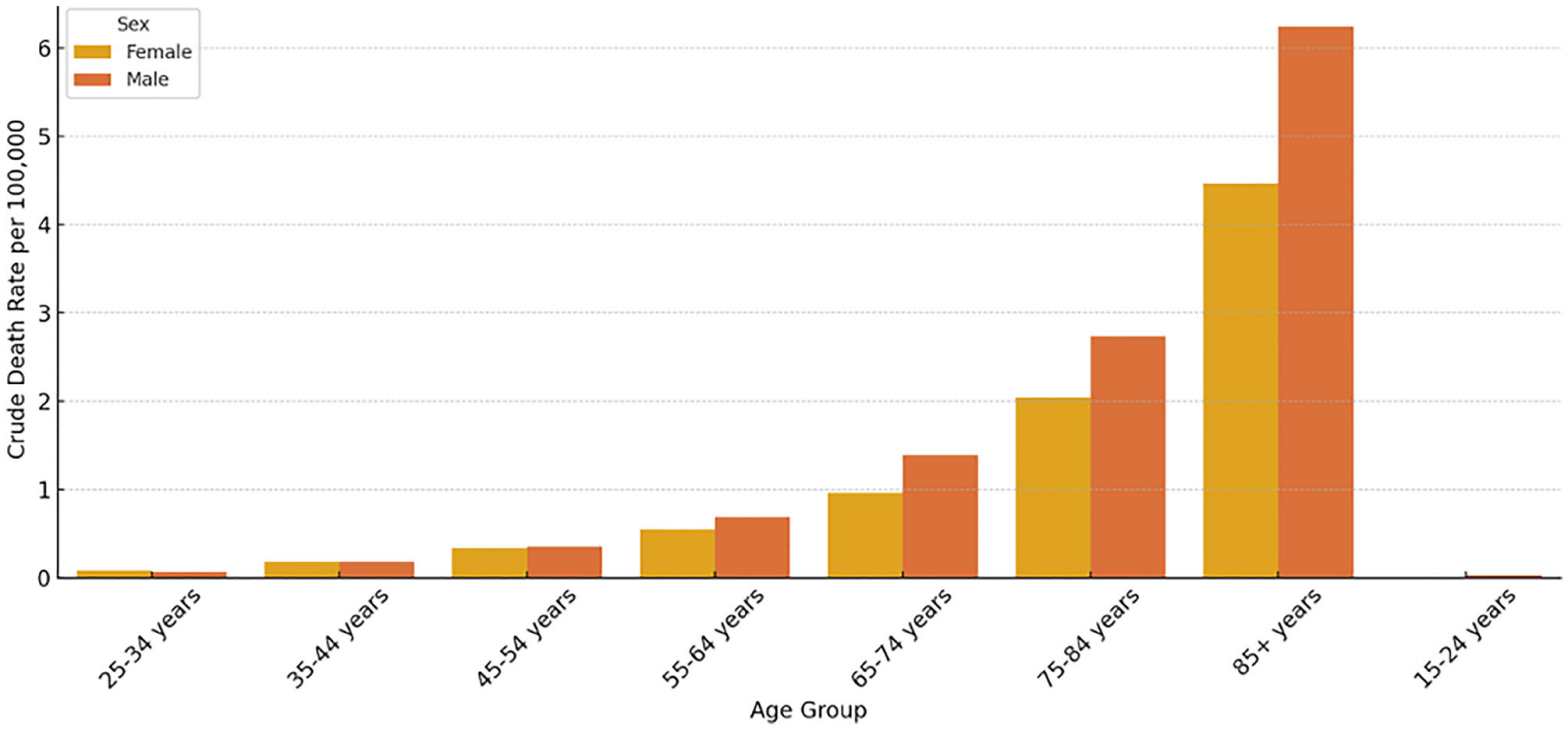
Crude death rate of SCD among CRC patients by sex and age group. Rates are presented per 100,000 population. Mortality increases progressively with age, with the highest burden observed among patients aged ≥85 years. Male patients consistently exhibit higher crude death rates compared to female patients across all age categories.

Figure 1 below shows that the crude death rates increased steadily with age in both sexes. Males consistently demonstrated higher death rates across all age groups, particularly notable from age 55 and above. The highest mortality burden was observed in the 65–74, 75–84 and 85+ age groups, aligning with known cardiometabolic risk increases in older CRC survivors.

### Trends

#### Overall trend

**Figure 2:**
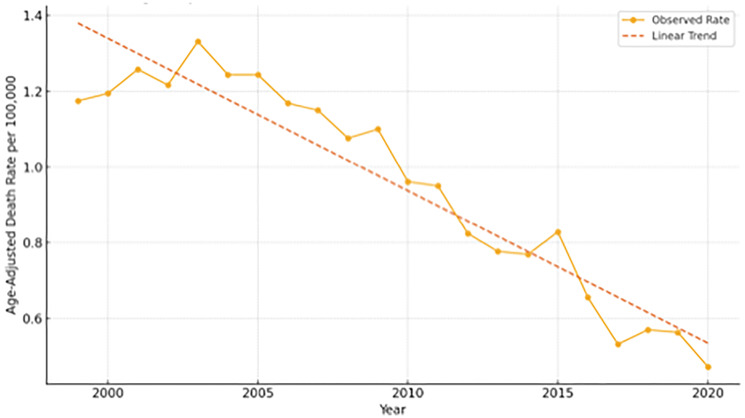
Age-adjusted death rate for sudden cardiac death (SCD) among colorectal cancer patients in the United States, 1999–2020. Rates are standardized to the 2000 U.S. standard population and expressed per 100,000 population. A significant linear decline is observed over the 21-year study period, decreasing from approximately 1.2 to 0.5 per 100,000, indicating a steady reduction in SCD-related mortality burden among colorectal cancer patients.

Rates were age-adjusted to the 2000 U.S. standard population and represented annual mortality per 100,000 population as depicted in Figure 2. A significant linear decline in mortality was observed (*p* < 0.001, R² = 0.897), from approximately 1.2 per 100,000 in 1999 to 0.5 per 100,000 in 2020. This trend indicated a steady reduction in SCD-related mortality burden among CRC patients over the study period.

#### Trends by sex

**Figure 3:**
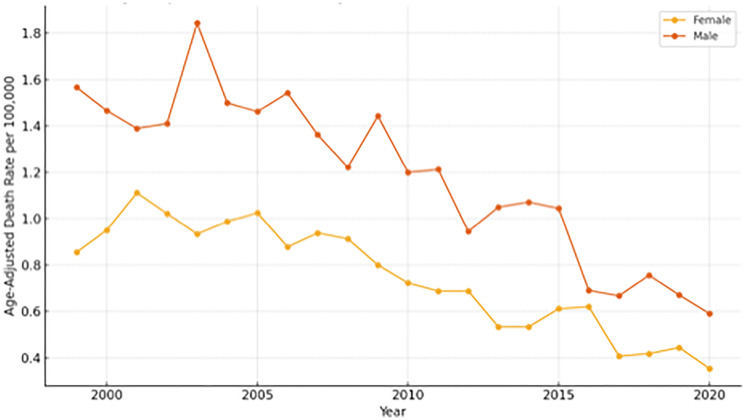
Age-adjusted death rates for sudden cardiac death (SCD) among colorectal cancer patients by sex in the United States, 1999–2020. Rates are expressed per 100,000 population and standardized to the 2000 U.S. standard population. Male patients consistently demonstrated higher mortality rates compared to females throughout the 21-year study period, although both groups experienced a general decline in mortality.

Figure 3 showed that male patients consistently exhibited higher mortality rates compared to females across the 21-year study period. Both groups experienced a general decline in mortality, although the reduction in females was more uniform.

#### Trend by race

**Figure 4:**
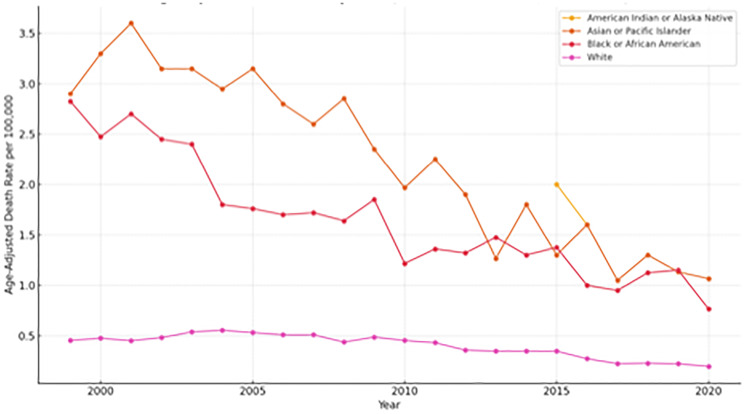
Age-adjusted death rates for sudden cardiac death (SCD) among colorectal cancer patients by race/ethnicity in the United States, 1999–2020. Rates are expressed per 100,000 population and standardized to the 2000 U.S. standard population. Although overall mortality declined across all racial groups during the study period, persistent disparities remained. Black or African American and Asian or Pacific Islander patients consistently had higher mortality rates compared to White patients. American Indian and Alaska Native patients demonstrated intermittent spikes, particularly in 2015, reflecting potential instability due to smaller population size but indicating a significant per-capita risk.

While all racial groups experienced a general decline in mortality over the 21-year period, persistent disparities remained. Asian or Pacific Islander and Black or African American patients consistently had higher mortality rates compared to White patients as shown in Figure 4. American Indian and Alaska Native patients showed intermittent but increased mortality spikes, particularly in 2015, indicating potential instability due to small population size but significant per-capita risk.

### Choropleth map

**Figure 5:**
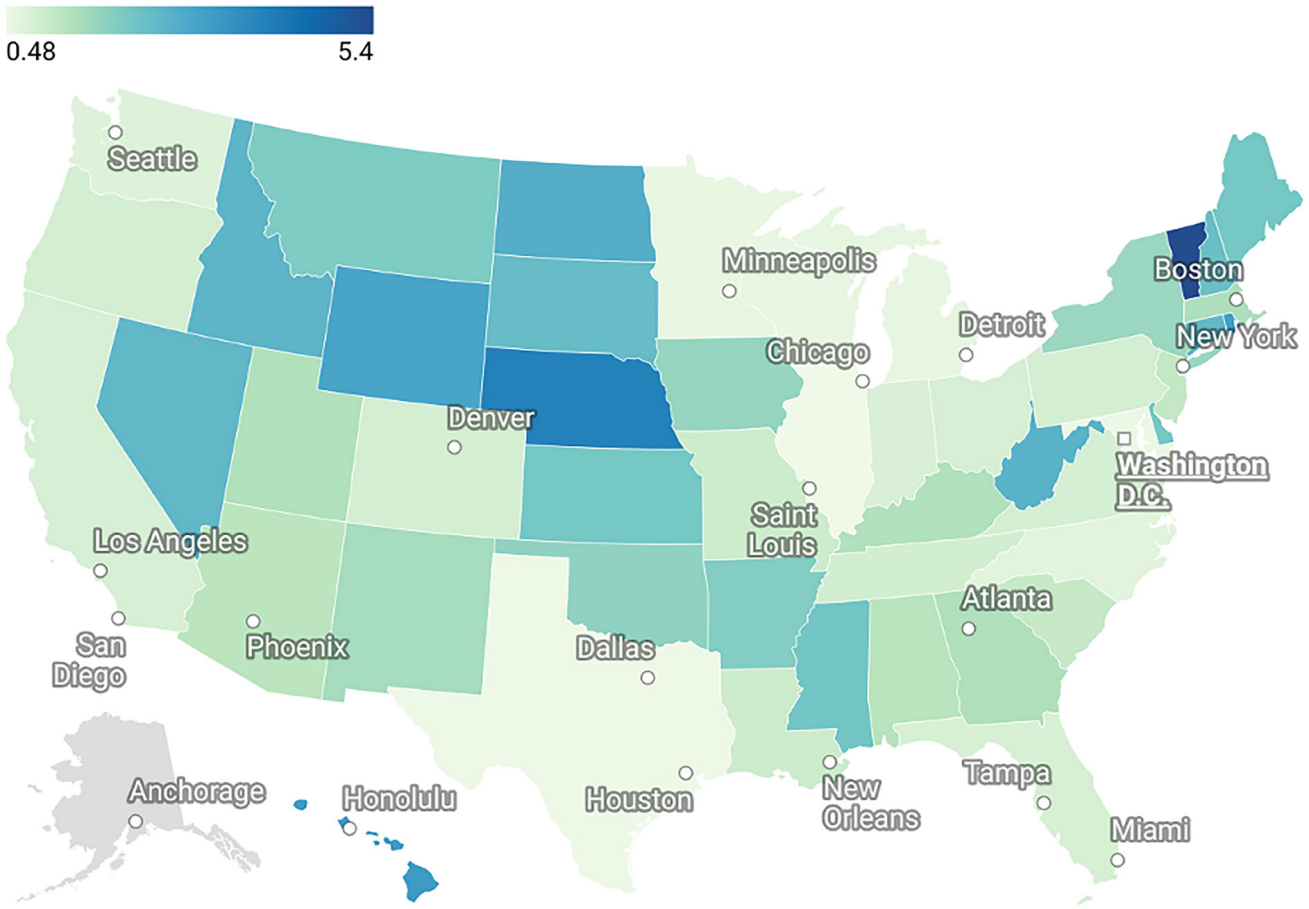
Crude death rates for sudden cardiac death (SCD) among colorectal cancer patients by United States, 1999–2020. Rates are expressed per 100,000 population. States such as Vermont, Nebraska, and Rhode Island show higher per-capita mortality burdens (darker shading), while larger states like California and Texas exhibit lower per-capita rates despite higher absolute case counts. States with insufficient data (e.g., Alaska) are shaded in gray.

The map presented in Figure 5 shows the state-level crude death rates (per 100,000 population) for sudden cardiac death (SCD) in patients with colorectal cancer (CRC), based on CDC WONDER data. States with higher rates (e.g., Vermont, Nebraska, Rhode Island) were shaded in darker blue, indicating a greater per capita burden. Populous states such as California and Texas showed lower rates due to population dilution despite high absolute case counts. States with missing data (e.g., Alaska) were shaded in gray.

## DISCUSSION

This study examined the rates of sudden cardiac death (SCD) among colorectal cancer (CRC) patients in the United States between 1999 and 2020, identifying many noteworthy trends. We found that crude death rates for SCD rose with age, with the highest burden in later age groups (65–84 years), and that male patients had consistently higher SCD mortality rates than females. Despite a considerable linear drop in age-adjusted SCD mortality during the research period, persistent and noticeable inequities were discovered, with Asian or Pacific Islander and Black or African American patients having persistently higher mortality rates than White patients. These findings broadly support trends and disparities reported in existing U.S.-based literature on cardiovascular outcomes and cancer survivorship, as well as broader global observations, indicating common pathophysiological mechanisms and demographic influences on SCD in this patient population.

Our finding that SCD crude death rates in CRC patients rise progressively with age, rising particularly in the 65–74 and 75–84 age groups, is consistent with national data on cardiovascular mortality, which regularly show greater incidence and mortality rates in older populations [15, 16]. This is further supported by studies focusing on cancer survivors, which show an increased risk of cardiovascular events, including SCD, as age increases after cancer diagnosis and treatment [6, 17]. Globally, the aging population and rising cancer incidence are recognized as important causes of cardiovascular disease burden in cancer survivors, including SCD [1, 18]. The cardiometabolic hazards associated with age, together with the systemic effects of cancer and its treatments, are likely to contribute to the observed trend on a global scale.

Male patients in our group had consistently higher SCD mortality rates than females, a tendency that has been reported in both the general population and cancer patients in the United States [19]. This gender gap is also evident in international literature, with various research from European, Asian, and other locations finding a higher prevalence of SCD in men [20, 21]. Previous research has shown that men tend to have greater numbers of traditional cardiovascular risk factors and may acquire cardiovascular disease at a younger age than women. While the precise mechanisms underlying this sex disparity in CRC-related SCD require further investigation, it could be due to inherent biological differences, a higher prevalence of cardiovascular risk factors in male CRC patients, or varying healthcare-seeking behaviors and adherence to secondary prevention strategies.

The significant linear decline in age-adjusted SCD mortality among CRC patients from 1999 to 2020 is indicative of broader advancements in colorectal cancer diagnosis and therapy, as well as improvements in cardiovascular disease prevention and treatment over the same period [22, 23]. This trend shows that better management of cancer and concomitant cardiovascular problems, such as enhanced screening, earlier detection, more effective CRC treatments, and advances in cardiology care, may contribute to a lower SCD burden in this vulnerable population. This is consistent with national health trends in the United States, which show lowering overall cardiovascular death rates [24], as well as comparable trends seen in many industrialized countries with enhanced healthcare infrastructure and public health programs [25]. We note that age-adjusted SCD mortality in the overall U.S. population has similarly decreased during the same period. The fall in CRC decedents from 1.2 to 0.5 per 100,000 is more significant than in the overall elderly population, where SCD rates remain higher (e.g., ~60 to 38 per 100,000 in those aged ≥65) [15]. This shows that CRC-specific cardiovascular management risk assessment, surveillance, and treatment planning may be benefiting this high-risk group.

Obesity, type 2 diabetes, sedentary behavior, and hypertension are among the many risk factors that overlap between colorectal cancer and sudden cardiac death [26]. These factors not only contribute to CRC etiology but also enhance the risk of lethal arrhythmias and ischemic cardiac events. Furthermore, the cardiotoxic properties of popular CRC therapies, particularly fluoropyrimidines, oxaliplatin, and antiangiogenic medicines, may exacerbate preexisting cardiovascular vulnerabilities [7]. As a result, the prevalence of SCD in CRC patients is likely due to both similar etiological pathways and treatment-related hazards, emphasizing the importance of coordinated cardio-oncology care. But at the same time, possible biases need to be recognized. Death certificate-based coding may not report or correctly describe SCD, especially when the death was sudden, or no one was there to see it. In addition, structural inequities, barriers to care access, and socioeconomic factors may make racial and geographic differences even bigger, which could make reported mortality gaps even bigger. These problems make it clear that the results need to be interpreted with care and stress how important it is to combine death records with prospective clinical cohort studies to confirm how SCD risk is increased in people with colorectal cancer.

Despite these overall declines, the persistent disparities observed, with Asian or Pacific Islander and Black or African American patients having consistently higher SCD mortality rates than White patients, are a critical finding that aligns with extensive literature on health inequities in the United States [27]. Numerous studies have found that racial and ethnic minorities have disproportionately higher rates of cardiovascular disease and worse outcomes, which can be attributed to a complex interplay of socioeconomic factors, systemic barriers to healthcare access, variations in the quality of care received, and a higher prevalence of underlying comorbidities [28]. While direct global comparisons of racial disparities in SCD among CRC patients are difficult due to differences in racial classifications and healthcare systems, international studies do highlight the impact of socioeconomic status and access to care on cardiovascular outcomes in cancer patients [1, 29].

Our research combined Asian and Pacific Islander populations, in line with CDC WONDER classification. This broad category could mask significant heterogeneity in SCD risk, as previous studies have demonstrated diversity in cardiovascular outcomes among subgroups. Future research using disaggregated datasets is necessary to reveal subgroup-specific discrepancies. Our findings also show that these systemic imbalances extend to the risk of SCD in CRC patients, underlining the critical need for focused interventions to achieve equitable health outcomes for all racial groups in this population. To address these gaps, different measures will be required, including community-based initiatives, greater access to preventative care, culturally competent healthcare delivery, and policies that address social determinants of health, both domestically and globally.

## MATERIALS AND METHODS

### Study design and data source

We conducted a retrospective, population-based mortality analysis using data from the Centers for Disease Control and Prevention (CDC) Wide-ranging Online Data for Epidemiologic Research (WONDER) Multiple Cause of Death (MCOD) database (https://wonder.cdc.gov/mcd.html) [15]. The MCOD dataset includes all U.S. death certificates from 1999 to 2020 and provides detailed information on underlying and contributing causes of death, as well as associated demographic and geographic variables.

### Study population

We identified decedents with colorectal cancer (CRC) listed as an underlying or contributing cause of death using ICD-10 codes C18–C21. Among these, we isolated cases in which sudden cardiac death (SCD) was also reported as a contributing cause using the following ICD-10 codes: I46.1 (sudden cardiac death, so described), I46.9 (cardiac arrest, unspecified), R96.0 (instantaneous death), R96.1 (death occurring less than 24 hours from onset of symptoms), I49.0 (ventricular fibrillation), and I21–I24 (acute myocardial infarction and related events).

### Variables extracted

We extracted and stratified data by year of death (1999–2020), sex (male, female), race/ethnicity (White, Black or African American, Asian or Pacific Islander, American Indian or Alaska Native), age group (ten-year intervals), state of residence, and corresponding population estimates. Age-adjusted death rates were standardized to the 2000 U.S. standard population. Race/ethnicity was extracted from CDC WONDER categories, which classify Asian and Pacific Islanders as a single group. This aggregation may obscure subgroup-specific differences (e.g., Chinese, Filipino, Vietnamese, Native Hawaiian).

### Outcome measures

The primary outcome was the SCD among CRC-related deaths. Secondary outcomes included subgroup analysis based on sex- and race-specific mortality patterns, and geographic variation across U.S. states.

### Statistical analysis

We used descriptive statistics to report total deaths and calculate crude death rates. Age-adjusted rates were extracted directly from CDC WONDER. Temporal trends in SCD mortality were assessed using linear regression. Differences in SCD mortality by sex, race. All analyses were conducted using Python, and a two-sided *p*-value <0.05 was considered statistically significant.

## Abbreviations

CRC: Colorectal Cancer
SCD: Sudden Cardiac Death
CDC: Centers for Disease Control and Prevention
WONDER: Wide-ranging Online Data for Epidemiologic Research
MCOD: Multiple Cause of Death
ICD-10: International Classification of Diseases, 10th Revision
IRB: Institutional Review Board
U.S.: United States
